# Brain circuits in autonomous sensory meridian response and related phenomena

**DOI:** 10.1098/rstb.2023.0252

**Published:** 2024-08-26

**Authors:** I-Fan Lin, Hirohito M. Kondo

**Affiliations:** ^1^ Department of Occupational Medicine and Clinical Toxicology, Taipei Veterans General Hospital, Taipei 112, Taiwan; ^2^ School of Psychology, Chukyo University, Nagoya, Aichi 466-8666, Japan

**Keywords:** autonomous sensory meridian response, sensory processing sensitivity, insula, salience network, default mode network

## Abstract

Autonomous sensory meridian response (ASMR) is characterized by a tingling sensation with a feeling of relaxation and a state of flow. We explore the neural underpinnings and comorbidities of ASMR and related phenomena with altered sensory processing. These phenomena include sensory processing sensitivity (SPS), synaesthesia, Alice in Wonderland syndrome and misophonia. The objective of this article is to uncover the shared neural substrates and distinctive features of ASMR and its counterparts. ASMR, SPS and misophonia exhibit common activations in the brain regions associated with social cognition, emotion regulation and empathy. Nevertheless, ASMR responders display reduced connectivity in the salience network (SN), while individuals with SPS exhibit increased connectivity in the SN. Furthermore, ASMR induces relaxation and temporarily reduces symptoms of depression, in contrast to SPS and misophonia, which are linked to depression. These observations lead us to propose that ASMR is a distinct phenomenon owing to its attention dispatch mechanism and its connection with emotion regulation. We suggest that increased activations in the insula, along with reduction in connectivity within the salience and default mode networks in ASMR responders, may account for their experiences of relaxation and flow states.

This article is part of the theme issue ‘Sensing and feeling: an integrative approach to sensory processing and emotional experience’.

## 1. Introduction

Autonomous sensory meridian response (ASMR) is a nonclinical term to describe a multidimensional phenomenon primarily triggered by auditory and audio-visual stimuli [1,2]. Individuals who experience ASMR describe a tingling sensation, originating in the head and spreading down to the body, accompanied by a feeling of relaxation and a mental state known as ‘flow’ [1,3]. In a flow state, a person is fully engrossed in an activity, free from self-consciousness, deeply focused and experiencing a distortion of time [4].

The estimated prevalence of ASMR ranges from 23.5 to 28% [5,6]. The common ASMR triggers include auditory and audio-visual stimuli made with persons whispering, eating, and using objects to make sounds [2]. Low-pitched sounds with dark timbre are effective in inducing ASMR [2,7].

ASMR can be regarded as an illusion or altered perception, transitioning from audio-visual information to tactile sensation. In this article, we compare ASMR with related sensory phenomena, including sensory processing sensitivity (SPS), synaesthesia, Alice in Wonderland syndrome (AIWS) and misophonia. Individuals with a SPS trait are easily overstimulated by external stimuli, and research has shown that their ASMR intensity is associated with SPS levels [8]. Synaesthesia is a phenomenon where a stimulus in one sensory modality elicits sensations in that modality and other modalities, and a higher prevalence of synaesthesia has been found among ASMR responders [1,6,9]. AIWS is a neurological condition known for distorted perception of the body and space, and it shares a distortion of time perception with ASMR. In addition, the prevalence of AIWS symptoms among ASMR responders (46.6%) is higher than the prevalence of AIWS in the general population (approx. 9%) [10–12]. Individuals with misophonia experience negative emotions and aversive responses elicited by specific auditory and visual stimuli. Given the overlap in triggers between ASMR and misophonia (e.g. eating sounds and repetitive tapping), ASMR and misophonia are suggested to share same mechanisms [13]. Although ASMR is linked to positive emotions, and misophonia is associated with negative emotions, there is a higher prevalence of misophonia among ASMR responders and *vice versa* [14–16].

This article reviews functional magnetic resonance imaging (fMRI) studies that investigate the brain activations observed in these phenomena or in individuals who experience these phenomena, with a focus on the following brain regions: the insula and cingulate cortex (CC) related to social cognition and emotion regulation, the nucleus accumbens (NAc) of the reward system, and the sensory-motor system, including the premotor area (PMA), supplementary motor area (SMA) and somatosensory cortex.

**Figure 1.**
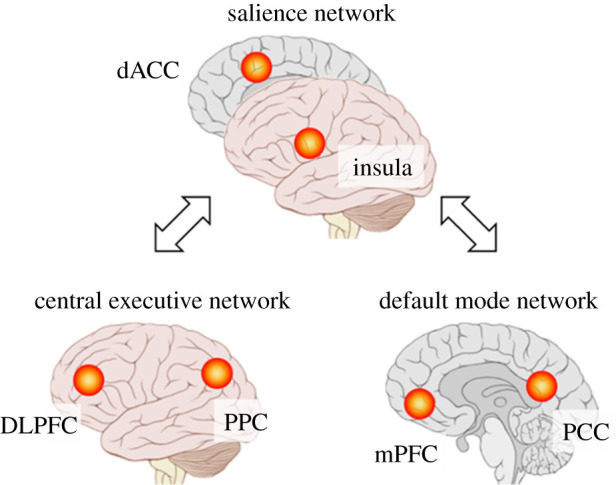
The framework of three large-scale networks in the human brain. The circles indicate important hubs in these networks. The salience network consists of the insula and dorsal anterior cingulate cortex (dACC). The central executive network includes the dorsolateral prefrontal cortex (DLPFC) and posterior parietal cortex (PPC), whereas the default mode network primarily comprises medial prefrontal cortex (mPFC) and posterior cingulate cortex (PCC).

Three large-scale brain networks in resting-state fMRI (rs-fMRI), including the salience network (SN), default mode network (DMN) and central executive network (CEN) (figure 1), are also compared between ASMR and its related phenomena. The SN is involved in detecting salient stimuli and switching between the DMN and the CEN [17]. The DMN is active when a person is at wakeful rest, such as during mind wandering and introspection. Meanwhile, the CEN is active when a person is engaged in specific tasks [18–22]. Through these comparisons, we aim to elucidate the similarities and differences in the brain circuits in ASMR and related phenomena. In addition to the brain circuits, we explore triggers, physiological responses, comorbidities and genetic predisposition associated with these phenomena.

To investigate brain activations and networks in these phenomena, we conducted a search on PubMed using keywords specific to each phenomenon, along with ‘fMRI’. In most cases, there are a handful of papers to review. Nevertheless, our search yielded numerous papers on synaesthesia. As many of these papers provide evidence supporting the theory of increased co-activations or connectivity across brain regions in synaesthetes, we only referred to some of the most cited papers that support and oppose this theory. Additionally, we searched for related literature using ‘electroencephalography (EEG)’ and ‘magnetoencephalography (MEG)’, but we found a very limited amount of literature using EEG and MEG to investigate these phenomena.

### (a) Autonomous sensory meridian response

ASMR elicits physiological responses in ASMR responders, including increased pupil diameters [23], enhanced skin conductance and reduced heart rate [24,25]. These observations suggest that ASMR induces arousal and relaxation among these individuals.

There are two types of fMRI studies in ASMR research: task-positive fMRI studies, which compare brain activations in ASMR responders and non-responders while they watch ASMR videos and control videos; and task-negative rs-fMRI studies, which compare brain functional connectivity between ASMR responders and non-responders. In task-positive fMRI studies, brain activations in the insula, dorsal anterior cingulate cortex (dACC), NAc, secondary somatosensory cortex and SMA are more pronounced for ASMR responders when they experience a tingling sensation during ASMR videos than watching a fixation cross (namely, the control condition) [26]. In another study, while both ASMR responders and non-responders watched ASMR and control videos, ASMR responders showed more activity in the dACC, PMA and SMA in the ASMR condition than in the control condition, but non-responders only showed increased activity in the cuneus [27]. Auditory-only ASMR stimuli have been shown to increase activations of the insula and NAc compared with the resting condition [28]. The involvement of the insula and ACC suggests the significance of social cognition, emotion regulation and the perception of social pain in ASMR experience [29,30]. The insula, ACC and NAc are also activated during music-induced chills [31–34].

While the activations in the insula and dACC, the key hubs in the SN, increase in ASMR responders when they watch ASMR videos, rs-fMRI studies showed less connectivity in the precuneus-centred DMN, SN and CEN (except in the left middle cingulate cortex) in ASMR responders than in non-responders [35,36]. Another study revealed higher functional connectivity seeded from the posterior cingulate cortex, a major hub of the DMN, to the superior and middle temporal gyri, cuneus, and lingual gyrus when comparing ASMR videos with resting state [37]. In addition to SN, DMN and CEN, other networks such as the sensorimotor and visual networks have been compared between ASMR responders and non-responders. The results show reduced functional connectivity in these two networks in ASMR responders [36].

The EEG studies exploring brain waves in different frequency ranges show increased alpha-wave activities when ASMR responders experience ASMR responses (i.e. tingling sensation) to ASMR stimuli [38,39]. Interestingly, footstep sounds, a less common ASMR trigger, were also observed to increased alpha-wave activities, especially among those who were less sensitive to these sounds and have a positive attitude toward them [40]. These findings indicate facilitation of attention-directed information and inhibition of irrelevant information [41]. This implication is consistent with the fMRI finding of insula activation in ASMR experiences, as the insula plays an important role in salience detection [17,42–44].

Although ASMR and autism share auditory sensitivity, there is no evidence supporting the association between ASMR and autism [45]. ASMR videos have been reported to alleviate symptoms of depression and anxiety [1,46].

### (b) Sensory processing sensitivity

SPS is defined as a personality trait characterized by being overstimulated by external stimuli, especially the positive ones, with heightened emotional responses [47,48]. SPS exhibits a prevalence ranging from 15 to 31% [48,49]. The Highly Sensitive Person (HSP) scale [49] has been used to measure the level of SPS, in addition to the Orienting Sensitivity subscale of the Adult Temperament Questionnaire [50]. The HSP scale comprises three subscales: low sensory threshold (LST), ease of excitation (EOE) and aesthetic sensitivity (AES) [51]. Genetic and twin studies suggest that SPS is influenced by genetic and environmental factors, with the AES subscale differentiating from the LST and EOE subscales [52,53]. The intensity of ASMR is associated with the AES subscale [8], while autism symptoms, alexithymia, anxiety and depression are linked to the EOE and LST subscales [54,55].

There are various triggers for individuals with SPS. In a study subjecting a group of adolescents to a stress test, their changes in heart rate, heart rate variability and skin conductance were not correlated with SPS, but their perceived stress was associated with SPS [56]. The relationship between SPS and subjective perception of stress indicates that the insula, especially the anterior insula, may play an important role in SPS. The anterior insula has been argued to integrate information from the internal and external stimuli, and its activity is associated with subjective feeling [42].

Several fMRI studies have explored the relationship between SPS levels and brain activations by using various stimuli. The SPS levels were positively correlated with reaction times to detect major and minor changes in visual stimuli and activations in the right claustrum, bilateral temporal cortex, bilateral precuneus and right inferior parietal cortex [57]. Another study observed the association between SPS levels and activations in the CC and PMA by using partner's and strangers’ faces with different emotions [58]. In addition, the primary somatosensory cortex and ventral tagmental area (VTA) were specifically activated by comparing the brain activations for partner's and strangers’ happy face. In another study, wherein the participants were touched by an experimenter, SPS levels were linked to brain activations in the insula [59]. Collectively, these findings in fMRI studies highlight the brain regions related to social cognition and empathy for social stimuli, such as the insula and CC, and those related to the reward pathway for positive stimuli, such as the VTA.

An rs-fMRI study has shown that the SPS levels were associated with enhanced brain connectivity within the SN and the dorsal attention network, as well as the limbic network [60]. These findings suggest an important role of attention and emotion regulation in SPS.

## 2. Synaesthesia

Synaesthesia is a phenomenon wherein a stimulus for a sensory modality elicits sensations in that modality and other modalities. The prevalence of various forms of synaesthesia is 4.4%, and the estimated prevalence of grapheme–colour synaesthesia, in which letters and numbers elicit colour perception, is 1.1% [61]. Although the presence of synaesthesia in family members suggests a hereditary connection [62,63], a twin study demonstrates that synaesthesia is a heritable condition that is influenced by epigenetic and environmental factors [64]. Previous studies have reported a higher prevalence of synaesthesia in the autism population compared with the neurotypical population. This association between autism and synaesthesia is believed to be mediated by attention to details and abnormal sensory processing [65–69].

Numerous studies have explored the neural basis of synaesthesia and identified activation in the sensory cortices beyond the stimulated modality and increased connectivity in sensory networks [70–73]. An rs-fMRI study revealed increased intranetwork connectivity from the medial and the lateral visual networks to the right frontoparietal network in grapheme–colour synaesthetes [74]. Nevertheless, ongoing debate persists about these findings and whether we should consider alternative neural models [75]. For example, the report of activation in the insula and CC in synaesthesia should be noted [76].

Mirror-touch synaesthesia, which involves experiencing tactile sensations on their own bodies upon seeing someone else touched [77], has been reported in approximately 1.6% of the population [78]. Some researchers debate about whether this phenomenon is a synaesthesia or vicarious experience, an empathetic state induced by seeing someone's sensations, emotions and actions [79]. Activations in the somatosensory cortex in mirror-touch synaesthetes may be related to empathy [80–83]. Additionally, mirror-touch synaesthetes exhibit higher autistic trait scores compared with the control group [84].

### (a) Alice in Wonderland syndrome

AIWS is a neurological condition characterized by distorted perception of the body, objects, space and time [10,85–87]. These perceptual distortions can manifest across the visual (e.g. dysmorphopsia, macropsia, micropsia and teleopsia), auditory and somaesthetic domains (e.g. macropsia, micropsia, teleopsia, macrosomatognosia, microsomatognosia and deceleration/acceleration of psychological time) [10,87,88] and have been observed in various clinical conditions, including migraine [88–90], epilepsy [91], infection [92–100] and concussion [101]. AIWS has also been found among people who use certain medication, such as 5-HT2 antagonist, and during intoxication with some substances, such as cannabis [10].

A lesion-mapping study identified local maxima across the lesions in the right occipital lobe, primarily driven by those with AIWS symptoms in the visual domain [102]. Those patients who had AIWS symptoms only in the somaesthetic domain exhibited lesions in the limbic system, including the insula, thalamus, CC, hippocampus and parahippocampus. This lesion study did not include participants perceiving time distortion, but the insula has been argued to be involved in time perception [103].

An imaging study that compared migraine patients with AIWS, migraine patients with typical aura, and healthy individuals showed that all migraine patients (with and without AIWS) had a lower functional connectivity in the lateral and the medial visual networks and higher functional connectivity in the SN and DMN compared with the healthy individuals [104].

## 3. Misophonia

Misophonia describes the phenomenon of experiencing negative emotions and aversive responses triggered by specific auditory and visual stimuli. Given the lack of well defined criteria for misophonia, estimates of its prevalence vary widely, ranging from 5.9 to 49.1% [16,105–109]. Although misophonia is not included in the *Diagnostic and statistical manual of mental disorders* (DSM), researchers have proposed diagnostic criteria of misophonia to address this issue [110–112]. Misophonia typically involves excessive and disproportional negative emotions, often leading affected individuals to avoid the triggering stimuli. Furthermore, individuals with misophonia exhibit physiological responses when exposed to such triggers, including increased skin conductance, elevated heart rate and an increased low-frequency component of heart rate variability [113–117]. These physiological responses indicate heightened sympathetic tones.

fMRI studies have established a connection between misophonia and increased brain activation in the insula, auditory cortex, temporal cortex, CC, medial prefrontal cortex (mPFC) and PMA. Furthermore, misophonia is linked to increased functional connectivity between the insula and the mPFC, the key hubs in the SN and the DMN [114,115,117], indicating the link between the SN and the DMN. On the other hand, the association between misophonia and increased brain activations in the orofacial motor area remains debated [118,119].

Misophonia has been found to be comorbid with various psychiatric conditions, including obsessive–compulsive personality disorder, mood disorders, attention-deficit hyperactivity disorder, autism, tinnitus, hyperacusis, post-traumatic stress disorder (PTSD), eating disorders, migraine with visual aura, anxiety sensitivity, and hypersensitivity [15,109,120–123].

## 4. Discussion

This review encompasses the identification of the common and distinctive neural circuits associated with ASMR and related sensory phenomena, including SPS, synaesthesia, AIWS and misophonia, to shed light on their underlying neural mechanisms. Among these phenomena, ASMR and SPS share activation in the reward pathway. Activation in the NAc are observed in ASMR, while activations in the VTA are observed in SPS. The involvement of these reward-related regions in ASMR is consistent with experiences of music-induced pleasure and frisson, characterized by the sensation of goose-bumps or shivers down the spine [33,34]. ASMR and mirror-touch synaesthesia share activation in the somatosensory cortex. While the role of the somatosensory cortex in mirror-touch synaesthesia is suggested to be linked to empathy, the role of the somatosensory cortex in ASMR may be linked to sound localization and head orientation [124,125].

The insula, a pivotal brain region, emerges as a shared activation site in ASMR, SPS, AIWS (in the somaesthetic domain) and misophonia. The insula has connections with the cingulate, frontal, parietal, temporal and occipital cortices and subcortical structures, such as the amygdala and NAc [126–129]. The insula plays an important role in novel stimuli detection and is part of the SN, alongside the dACC and amygdala. In addition to salience detection, the insula is involved in visceral sensations, autonomic control, affective processes, sensorimotor processes, auditory processing, taste, olfaction and pain. The insula is proposed to be the centre of awareness and the interoceptive template for a ‘feeling’ [42–44]. The insula and ACC are closely connected and usually activate together, and they play an important role in social pain and empathy [130,131]. In summary, activation in the insula observed in ASMR, SPS, AIWS and misophonia is associated with peculiar perception and emotional responses observed in these phenomena.

Diverging from the previously mentioned shared brain activations, rs-fMRI studies uncover unique facets of these phenomena. ASMR responders exhibit weaker connectivity in the SN despite strong activations in the key hubs of the SN, the insula and the dACC. This finding suggests a diminished capacity for attention switch between internal and external stimuli. Furthermore, non-synchronization of the SN with strongly activated insula and dACC may result in altered perception. By contrast, increased connectivity in the SN is observed in SPS and AIWS, potentially linked to their sensory sensitivity or distortion.

This review also explores the comorbidities associated with these phenomena. ASMR exhibits links to temporary reduction of anxiety and depression symptoms but not to autism. Meanwhile, SPS is associated with autism, anxiety and depression; synaesthesia with autism; AIWS with migraine; and misophonia with autism, anxiety, depression and migraine. In other words, ASMR is on the opposite of the phenomena SPS, synaesthesia and misophonia.

This review acknowledges certain limitations. Heterogeneity amongst the participants in ASMR, SPS, synaesthesia, AIWS and misophonia studies, stemming from symptom-based definitions and questionnaire assessments poses a significant limitation. The modest amount of literature on these phenomena (except synaesthesia) also exacerbates this limitation. Furthermore, the diversity of the stimuli employed in physiological and imaging studies, the relatively small sample sizes and variations in experimental paradigms contribute to the need for caution in drawing definitive conclusions. Accordingly, this article serves as a basis for further investigations aimed at unravelling the intricate correlations between ASMR-induced sensations and neural networks. Future studies should address standardized stimuli and psychophysical approaches to objectively identify the target group.

## 5. Conclusion

The culmination of previous studies demonstrates that ASMR is distinct from related phenomena with altered sensory processing, including SPS, synaesthesia, AIWS and misophonia. Nonetheless, ASMR, SPS and misophonia all involve activation of the insula and CC related to social cognition and emotion regulation. ASMR differentiates itself from SPS through its reduced connectivity within the SN. Reduced connectivity in the SN during ASMR experiences indicates a particular way to direct attention that links to the feeling of relaxation and the flow state.

## Data Availability

All data gathered for this review may be available upon request to the corresponding author.
